# Supplementary material for: Quercetin enhances motility in aged and heat-stressed Caenorhabditis elegans nematodes by modulating both HSF-1 activity, and insulin-like and p38-MAPK signalling
Source: PLoS One. 2020 Sep 3;15(9):e0238528. doi: 10.1371/journal.pone.0238528 (PMC7470330; doi:10.1371/journal.pone.0238528)
Supplement: S1 File — (DOCX) [file pone.0238528.s001.docx]

Supplemental data

EGCG (Cayman) was dissolved in dimethyl sulfoxide (DMSO; Kanto Chemical) to a concentration of 0.02 mg/ml (43.6 µM), before being diluted to 0.1- and 1.0 mg/ml (0.22 ,2.2 µM respectively) in OP50, and spread onto NGM plates for each assay. We defined the plates spread onto NGM plates with EGCG and OP50 as EGCG plate. 200 µl of OP50 and EGCG were spread onto NGM plates and dried.

*Evaluation of motility in ageing nematodes*

Synchronized worms were cultured on OP plates at 20°C for 96 h, and then transferred to OP, Q plates (500 µM) or EGCG plates (0.1, 1.0 mg/ml) (day 0) for further culture (20°C). The movement of worms was counted until day 12. Other manipulations are same to what written in materials and methods.

*Evaluation of nematode motility recovery after heat stress*

To measure the motility-recovery rate after heat stress, synchronized wild type worms were cultured on OP, Q plates (500 µM) or EGCG plates (0.1, 1.0 mg/ml) at 20°C for 96 h. Heat-stress initiation was designated as 0 h, and the thrashing movements in 15 seconds of both heat-stressed and control (maintained at 20°C) worms were counted every 6 hours in S-basal medium. Other manipulations are same to what written in materials and methods.

*Gene expression*

Synchronized worms were cultured on OP, Q plates (500 µM) or EGCG plates (0.1, 1.0 mg/ml) for 96 h. Primer list is on a table (S1 Table). Other manipulations are same to what written in materials and methods. Primers are written in the table (S1 Table)

S1 Table

| **Gene** | **Sense primer (5’–3’)** | **Antisense primer (5’–3’)** |
| --- | --- | --- |
| *actin* | TCGGTATGGGACAGAAGGAC | CATCCCAGTTGGTGACGATA |
| *tba-1* | TCAACACTGCCATCGCCGCC | TCCAAGCGAGACCAGGCTTCAG |
| *pmp-3* | TGGCCGGATGATGGTGTCGC | ACGAACAATGCCAAAGGCCAGC |
