# Supplementary figures and images for: Quercetin enhances motility in aged and heat-stressed Caenorhabditis elegans nematodes by modulating both HSF-1 activity, and insulin-like and p38-MAPK signalling
Source: PLoS One. 2020 Sep 3;15(9):e0238528. doi: 10.1371/journal.pone.0238528 (PMC7470330; doi:10.1371/journal.pone.0238528)

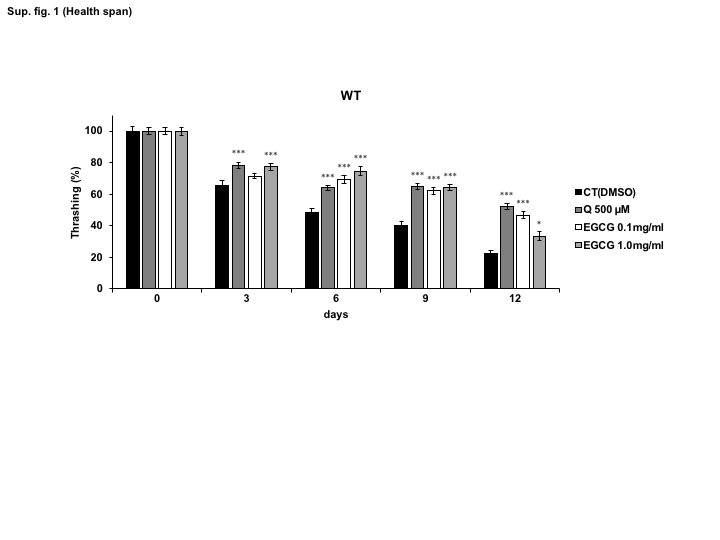

Supplement: S1 Fig — Movement counts were generated for N2 Caenorhabditis elegans nematodes every 3 days from day 0 until day 12, and presented relative to that calculated on day 0. Data are presented as the mean ± SEM, n = 10/group. *P < 0.05, ***P < 0.005 vs CT according to the conducted Tukey’s HSD. All assays were conducted at least three times independently. (TIFF) [file pone.0238528.s002.tiff]

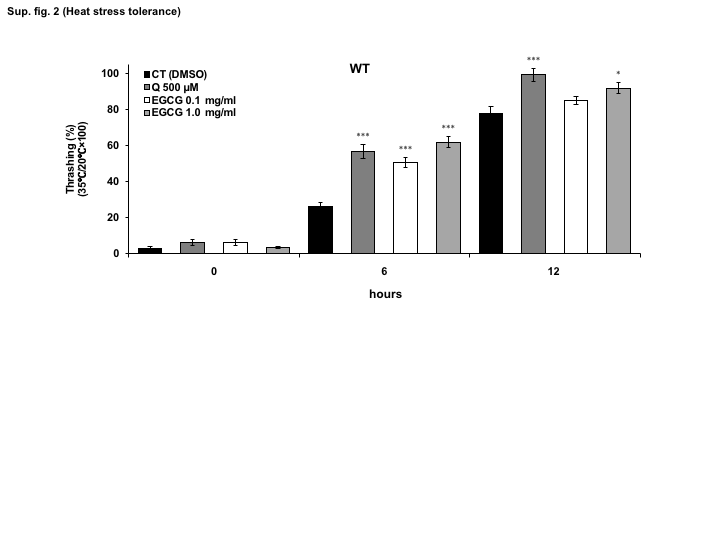

Supplement: S2 Fig — Movement counts were generated to assess the motility-recovery rate of N2 Caenorhabditis elegans nematodes at 6 h intervals after heat stress. Data are presented as the mean ± SEM, n = 10/group. *P < 0.05, ***P < 0.005 vs CT (DMSO) according to the conducted Tukey’s HSD. All assays were conducted at least three times independently. (TIFF) [file pone.0238528.s003.tiff]

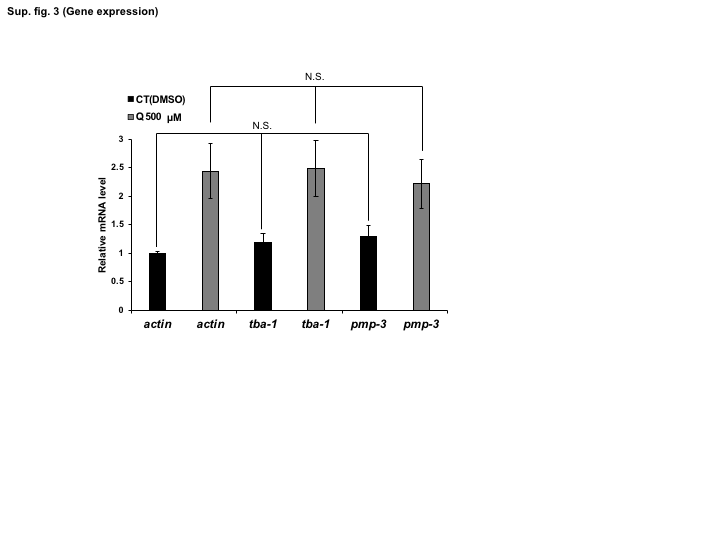

Supplement: S3 Fig — The mRNA expression levels of the analysed genes are shown relative to that of the quantity of actin in CT. Data are presented as the mean ± SEM. The quantities of genes in each condition are not significantly. Assays were conducted at least three times independently. (TIFF) [file pone.0238528.s004.tiff]
