# Supplementary material for: Quercetin enhances motility in aged and heat-stressed Caenorhabditis elegans nematodes by modulating both HSF-1 activity, and insulin-like and p38-MAPK signalling
Source: PLoS One. 2020 Sep 3;15(9):e0238528. doi: 10.1371/journal.pone.0238528 (PMC7470330; doi:10.1371/journal.pone.0238528)
Supplement: Attachment — Submitted filename: Replies to reviewers-QCN-TS-Final.docx [file pone.0238528.s005.docx]

**Replies to reviewers' comments**:

*All corrections are shown in red in the text.

**Reviewer #1**

1. Line 69: Please delete the word of “signaling” at the period

<Reply 1>

We appreciate to the reviewers’ comment. Following the reviewers’ suggestion, we deleted the word of “signaling”

See -🡪 P4, L69

2. Please describe in detail of OP or Q plates

<Reply 2>

OP plate and Q plate are the plates coated with *E. coli* (OP50) and quercetin (50µl, 500µl), respectively, onto a NGM plate as written in Materials and methods.

See -🡪 P5, L80

3. Please point the detail concentration of OP and O in each experiment

<Reply 3>

Basically we used the concentration 50µl and 500µl only. We described the concentration in Materials and methods.

See -🡪 P7, L96 and 107, P8, L122, P9, L134 and 142, P10, L150

4. Line 224-227: the authors showed that the heat stress disrupts nematode proteostasis by causing aberrant protein folding and aggregation in a manner similar to aging, so the motility of heat-stressed worms is considered to be equivalent to that of aged worms, and to be representative of the nematode healthspan. Please list related references

<Reply 4>

We appreciate to the reviewers’ comment. Following the reviewers’ suggestion, we inserted two references [33, 34].

[33] Ravi Raghav Sonani. *et al*. Phycoerythrin extends life span and health span of *Caenorhabditis elegans*.

Age (Dordr). 2014;36(5):9717. doi: 10.1007/s11357-014-9717-1.

[34] Philippe Verbeke, *et al.* Heat Shock Response and Ageing: Mechanisms and Applications.

Cell Biology International 2001, Vol. 25, No. 9, 845–857.

See -🡪 P15, L239

5. It is recommended offer the data of the positive control in each experiment

<Reply 5>

We appreciate to the reviewers’ comment. Following the reviewers’ suggestion, we inserted one reference [5] and supplemental data using EGCG, as a positive control.

[5] Zhang, L. *et al*. Significant longevity-extending effects of EGCG on Caenorhabditis elegans under stress. *Free Radical Biology and Medicine*, *46*(3), 414-421 (2009).

See -🡪 P3, L42, P12, L185, P13, L202, P15, L241 and in supplemental data

**Reviewer #2**

Obtained data on quercetin are not consistent with the results of other model studies (<https://www.ncbi.nlm.nih.gov/pmc/articles/PMC5179547/>) and there is not enough discussion of the reasons for such differences.
<Reply 1>

As written in the previous paper [13], low concentration of quercetin has positive effects, whereas high concentration has negative effects. The effects on aging phenotypes might be different depending on the concentration of quercetin and animals to apply.

[13] Proshkina, E. *et al*. Geroprotective and Radioprotective Activity of Quercetin, (-)-Epicatechin, and Ibuprofen in *Drosophila melanogaster*. *Front. Pharmacol*. **7**, 505 (2016).

See -🡪 P3, L46

Only one house-keeping gene as a reference in RT-PCR, but actin has age-related changes in its expression. It is better to try 2-3 different house-keeping genes.

<Reply 2>

We performed qPCR assay using *tba-1* and *pmp-3* as the reference genes recommended in a paper [31], and inserted these data and one reference [31] into text. Actin could be a proper reference gene because the quantity of mRNA expression of actin, *tba-1* and *pmp-3* didn’t change significantly.

[31] Zhang, Y *et al*. Selection of reliable reference genes in *Caenorhabditis elegans* for analysis of nanotoxicity. PloS one 7.3 (2012): e31849.

See -🡪 P10, L156, P14, L219 and in supplemental data

The authors have to carefully check the style. For example the word "modulate" used 4 times in the abstract.
In a row 69: signaling [4,14-16]. Signaling

<Reply 3>

We appreciate to the reviewers’ comment. Following the reviewers’ suggestion, we replaced the words “modulate” and “signalling” to some others or removed and abbreviate insulin-like signaling as ILS.

See -🡪 P2, L28-35, P4, L68, P5, Table1, P16, L250, 256-259, P17, L271 and

273 and P19, L305
